# Weight loss magnitude, prevalence and methods among male and female Olympic-level judo athletes

**DOI:** 10.1186/s13102-025-01478-8

**Published:** 2025-12-16

**Authors:** Ayşe Feray Özbal, Arıkan Ektirici, Bayram Ceylan

**Affiliations:** 1 Department of Physical Education and Sports, Faculty of Sport Sciences, Kastamonu University, Kastamonu, Türkiye; 2 Department of Coaching Education, Faculty of Sport Sciences, Kastamonu University, Kastamonu, Türkiye; 3 Faculty of Sport Sciences, Kastamonu University, Kastamonu, 37150 Türkiye

**Keywords:** Rapid weight loss, Weight loss behaviors, Weight regain, Weight loss

## Abstract

Weight loss (WL) is common among judokas; however, most studies concentrate on non-elite competitors. This study examined the extent, methodologies, and contributing variables of WL among Olympic-level judo competitors during the qualifying cycle, positing that elevated WL scores signify detrimental practices. Research data was collected using the cross-sectional survey method. Research data was collected from 44 judokas (32 males, 12 females) rated in the top 50 globally during 2024 Antalya Grand Slam. Independent samples t-tests and Mann-Whitney U tests were used to assess between-group differences, while Spearman’s rank-order correlation coefficients were calculated to assess the relationship between variables. Male athletes had markedly elevated typical weight loss percentages, peak career weight reduction (in kg and %), and post-competition weight return compared to females. Gradual dieting (87.9%), meal skipping (77.3%), increased exercise (72.7%), and sauna usage (70.5%) were the most common WL methods. The main sources of influence were coach/sensei (70.5%), fellow judokas (50%), and peers in the judo community (47.7%). Health professionals had little effect on WL behaviors. The age of judo practice onset was correlated with higher weight loss percentages and weight regain. Olympic-level judokas participate in substantial WL, mostly shaped by their coaching and peer dynamics. The results indicate that coaches and players need to have focused information to encourage safer ways to control their weight.

**Supplementary Information:**

The online version contains supplementary material available at 10.1186/s13102-025-01478-8.

## Introduction

Judo is an Olympic combat sport where athletes compete according to weight categories [1]. The aim of the weight categories is to make an environment where athletes do not gain any advantage against their opponents and minimize the risk of injury [2, 3]. However, a considerable number of judo athletes, ranging from 70 to 90%, from different age and competitive level resort to weight loss (WL) and rapid weight gain (RWG) a few days preceding the official competitions using pathogenic methods [3–7]. The most preferred methods by judo athletes include reducing food and fluid intake, increasing exercise with additional clothing and heated environments [3, 5, 8].

These pathogenic methods by the athletes who are above their weight classes [9] may lead to impaired performance and put the athletes’ health at risk [10]. WL led to impaired repeated aerobic and anaerobic performance and decreased strength [11–13]. Ceylan, Aydos [14] investigated effect of rapid WL on hydration status and judo specific performance and stated WL aggravated hypohydration and increased cardiovascular load during repeated judo-specific performance. Moreover, WL is associated with long-term health problems including eating disorders, obesity, impaired growth, and high risk of cardiovascular disease [11, 15, 16]. Given that judo athletes start WL at a very early age [3, 4] and resort to WL very often [3, 8] and with great magnitude [7] regardless of competition rankings, they may be at high risk of health problems.

The existing literature on weight management in combat sports often highlights sex-based differences in WL behaviors. Previous studies across disciplines such as wrestling, boxing, and judo suggest that male athletes typically engage in more frequent and severe weight-cutting practices, often resorting to more extreme methods like dehydration [17, 18], while female athletes may report a higher prevalence of dieting and chronic weight control measures [17, 19, 20]. Despite numerous studies related to WL among judo athletes, existing studies primarily focused on amateur, regional or national-level judo athletes [3, 4, 21] or those in the first 150 of each weight category [8]. Presenting weight loss habits of Olympic-level judo athletes are particularly notable as they face unique pressure during Olympic qualification period during which they have to maintain peak physical condition [22] and adhere to strict weight class requirement while participating in as many tournaments as possible to qualify and collect the highest points to be seeded in the world ranking list to avoid the best athletes in the eliminations [1]. The Olympic Games are the summit of athletic achievement, and the methods used by these top judo athletes may differ dramatically from their non-Olympic counterparts, both in terms of complexity and potential risk. This study intended to evaluate weight loss habits of athletes who are very close to be qualified for the Olympic Games, thus exposing to numerous competitions to be seeded, trying to keep their ranking or qualifying for the Games. Therefore, this study aimed to investigate WL habits of Olympic-level judo athletes, presenting magnitude, prevalence and methods of WL during Olympic qualification cycle. We believe this study will contribute to the field by shedding light on the weight loss behaviors of this unique population and providing useful data for coaches and researchers. This study hypothesized that Olympic-level judo athletes would present high WL scores, indicating pathogenic WL habits and there would be differences in weight loss variables between male and female athletes.

## Materials and methods

### Study design

Research data was collected using cross-sectional survey method. A cross-sectional survey collects information from a sample that has been drawn from a predetermined population [23]. The data was collected during 2024 Antalya Grand Slam.

### Procedures

After participants approved to participate in the study, the researchers informed the athletes related to the nature of study and the questionnaire used. Then each item in the questionnaire was answered by the participants with the help of researchers. Participants were informed that the data would be kept anonymous to ensure that they provided honest answers to the survey. In addition, researchers were available to participants during the data collection process to provide extensive information related to questionnaire items and answer any questions they had throughout the procedure.

### Rapid weight loss questionnaire

WL methods among judokas were determined using a WL questionnaire developed by Artioli, Scagliusi [9] (English translation was already published by the researchers). This self-reporting questionnaire consists of 21 items related to age, height, weight, competitive level, WL history, source of influence on weight loss practices (e.g. another judoka, sensei, physical trainer, dietitian etc.) and WL methods (e.g. skipping meals, fasting, laxatives, diet pills etc.).

### Participants

The sample consisted of 44 judokas, of which 32 males (72.7%) and 12 females (27.3%), who are in the top 50 in the world ranking in their weight category according to IJF. Average age of the sample was 24.15 years (SD: ±4.18) for the males and 21.41 years (SD: ±4.14) for the females while the mean height was 171.28 cm (SD: ±7.97) for the males and 162.91 cm (SD: ±6.81) for the females. The mean weight was 74.33 kg (SD: ±10.88) for the males and 58.85 kg (SD: ±6.62) for the females. The average age to start practicing judo was 7.34 years (SD: ±2.28), whereas the mean age to start participating in judo competitions was 9.82 years (SD: ±2.51). Furthermore, both male and female participants’ average off-season weight was 71.26 (SD: ±12.48).

The study included participants from 20 countries including Türkiye (27.3%), Canada (9.1%), Belgium (6.8%), Great Britain (6.8%), United States (6.8%), Spain (4.5%), Finland (4.5%), Portugal (4.5%), Slovenia (4.5%), Australia (2.3%), Croatia (2.3%), Estonia (2.3%), Israel (2.3%), Estonia (2.3%), Korea (2.3%), Maldives (2.3%), Poland (2.3%), Switzerland (2.3%), Slovakia (2.3%), Sweeden (2.3%), and Taiwan (2.3%).

The research was carried out in accordance with the latest version of Helsinki Declaration, and ethical approval was acquired from the Non-Interventional Clinical Research Ethics Committee of the Kastamonu University (Ref. No KAEK-143-54). All athletes signed written informed consent form before agreeing to participate. Since one of the participants was under the age of 18 (minor), his/her national team coach provided formal approval as his/her legal guardian during the data collection process.

### Statistical analysis

Obtained data was analyzed using SPSS (version 20.0). The mean and standard deviation were utilized for presenting continuous variables, whereas frequencies (%) were used for demonstrating categorical variables. The Shapiro-Wilk test was utilized to check the normality of the data. Highest weight loss in career (kg), highest weight loss percentage (%), weight regains after one week of competition (days), age began weight loss (years), the number of days spent losing weight before the competition (days), were analyzed using Mann-Whitney U, whereas usual weight loss (kg), usual weight loss percentage (%), and WL total scores were analyzed using Independent Samples t-Test, depending on gender. For the Independent Samples t-Tests, Cohen’s d was calculated to determine whether the effect sizes (d < 0.20), (0.21–0.60), (0.61–1.20), (1.21–2.00), (2–4), and (> 4) were very small, small, moderate, large, very large, or nearly perfect (Cohen, 1988). For the Mann-Whitney U tests r (effect size) was calculated to determine the effect sizes (*r* = 0.10), (*r* = 0.30), (*r* = 0.50) were small, medium or large (Cohen, 1992, p.157). The correlation between the age at which participants started practicing judo, WL total scores, highest weight loss percentage (%), usual weight loss percentage (%), highest weight loss in career, usual weight loss values, and weight regains after one week of competition were calculated by Spearman’s Rank Correlation Coefficient. Spearman test values (ρ) (≥ 0.70), (0.40–0.69), and (0.1–0.39) were interpreted as strong relationship, moderate relationship, and weak relationship, respectively [24]. The level of significance was set at *p* < 0.05.

## Results

Independent samples t-test results are shown in Table 1. There was no significant difference between sexes in terms of weight loss value (t_44_ = 1.68, *p* > 0.05) and WL total scores (t_44_ = 0.74, *p* > 0.05). However, usual weight loss percentages of male (x̄=2.91) judokas were statistically higher than female (x̄=1.76) judokas (t_44_=2.97, *p* < 0.05, d = 1.15, [moderate]).

**Table 1:** Independent samples t-Test results according to WL history

	Group	Mean	SD	t	*p*
Usual weight loss value (kg)	Male	3.90	1.46	1.68	0.10
	Female	3.08	1.37		
Usual weight loss percentage (%)	Male	2.91	1.25	2.97	0.00*
	Female	1.76	0.66		
WL total score	Male	43.58	9.57	0.74	0.46
	Female	41.27	8.15		

*p<0.05, SD=standard deviation

Mann-Whitey U results are shown in Table 2. The findings of the analysis indicated that the variables of participants’ age at which they began weight loss (U = 160.50, *p* > 0.05) and the number of days spent losing weight before competition (U = 151.50, *p* > 0.05) did not significantly differ according to gender. Conversely, male judokas displayed higher mean rank values compared to female judokas in the following variables: highest weight loss in career (U = 117.50, *p* < 0.04, *r* = 0.29, [small]), highest weight loss percentage (U = 71.50, *p* < 0.05, *r* = 0.47, [medium]), and weight regains after one week of competition (U = 111.00, *p* < 0.05, *r* = 0.33, [medium]).

**Table 2:** Mann-Whitney U results according to WL history

	Group	Mean Rank	Sum of Ranks	U	*p*
Age began weight loss (years)	Male	23.48	751.50	160.50	0.40
	Female	19.88	238.50		
Highest weight loss in career (kg)	Male	24.83	794.59	117.50	0.04*
	Female	16.29	195.50		
Highest weight loss percentage (%)	Male	26.27	840.50	71.50	0.00*
	Female	12.46	149.50		
Weight regains after one week of competition (kg)	Male	25.03	801.00	111.00	0.02*
	Female	15.75	189.00		
The number of days spent losing weight before the competition (days)	Male	21.23	679.50	151.50	0.28
	Female	25.88	310.50		

*p<0.05

**Fig. 1:**
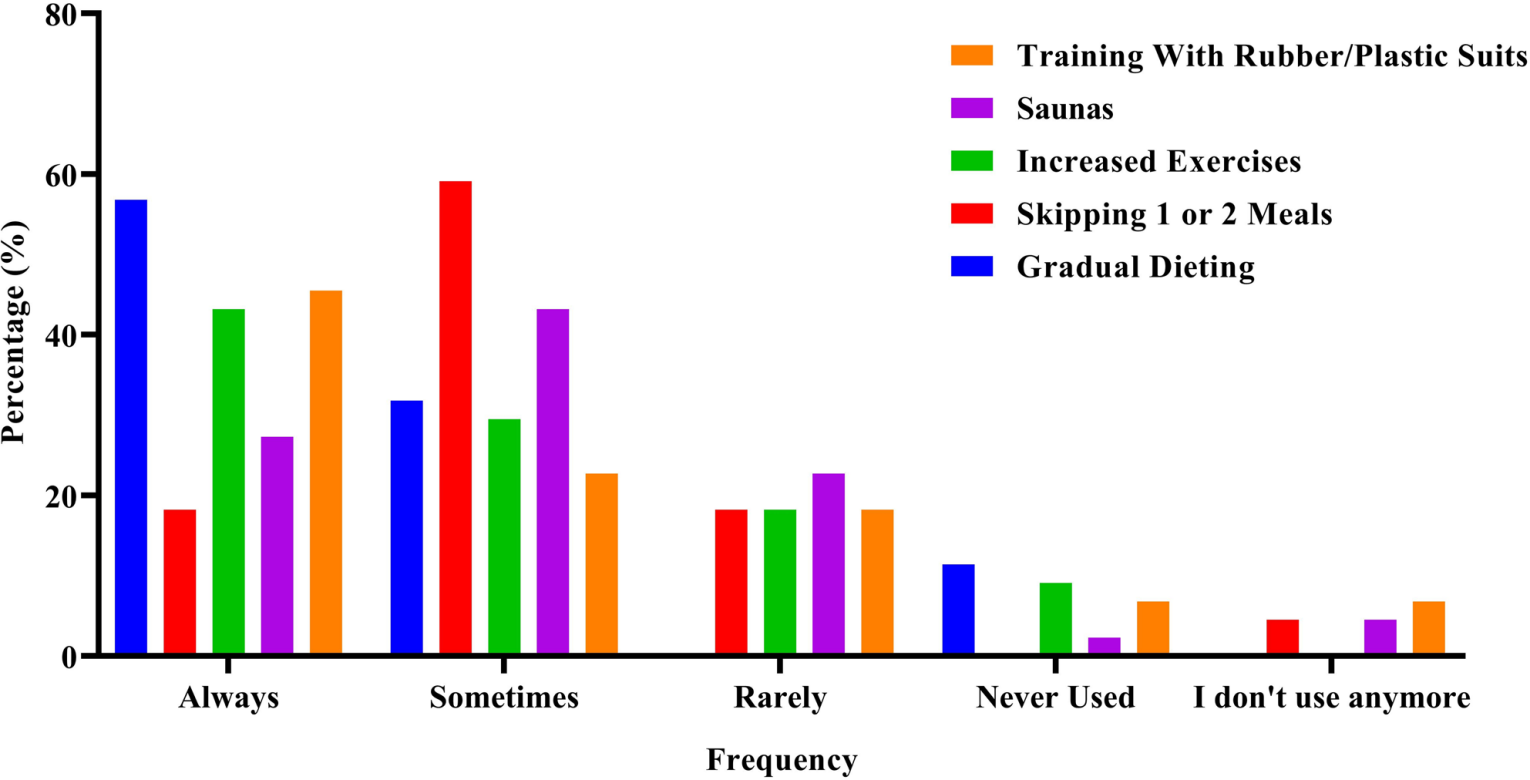
Frequency distribution (%) of the most used WL methods preferred by judokas

The predominant methods employed for WL (quantified as a sum of responses “always” and “sometimes”) included gradual dieting (87.9%), skipping 1 or 2 meals (77.3%), increased exercises (72.7%), saunas (70.5%), training with rubber/plastic suits (68.2%), training in intentionally heated rooms (59.3%), restricting fluid ingestion (56.8%), and fasting (52.3%). The less prevalent methods used included laxatives (34.0%), using winter or plastic suits whole day (29.6%), diuretics (20.5%), vomiting (20.5%), diet pills (15.9%), and spitting (13.6%) (see Fig. 1 and Tables in the supplementary file).

**Fig. 2:**
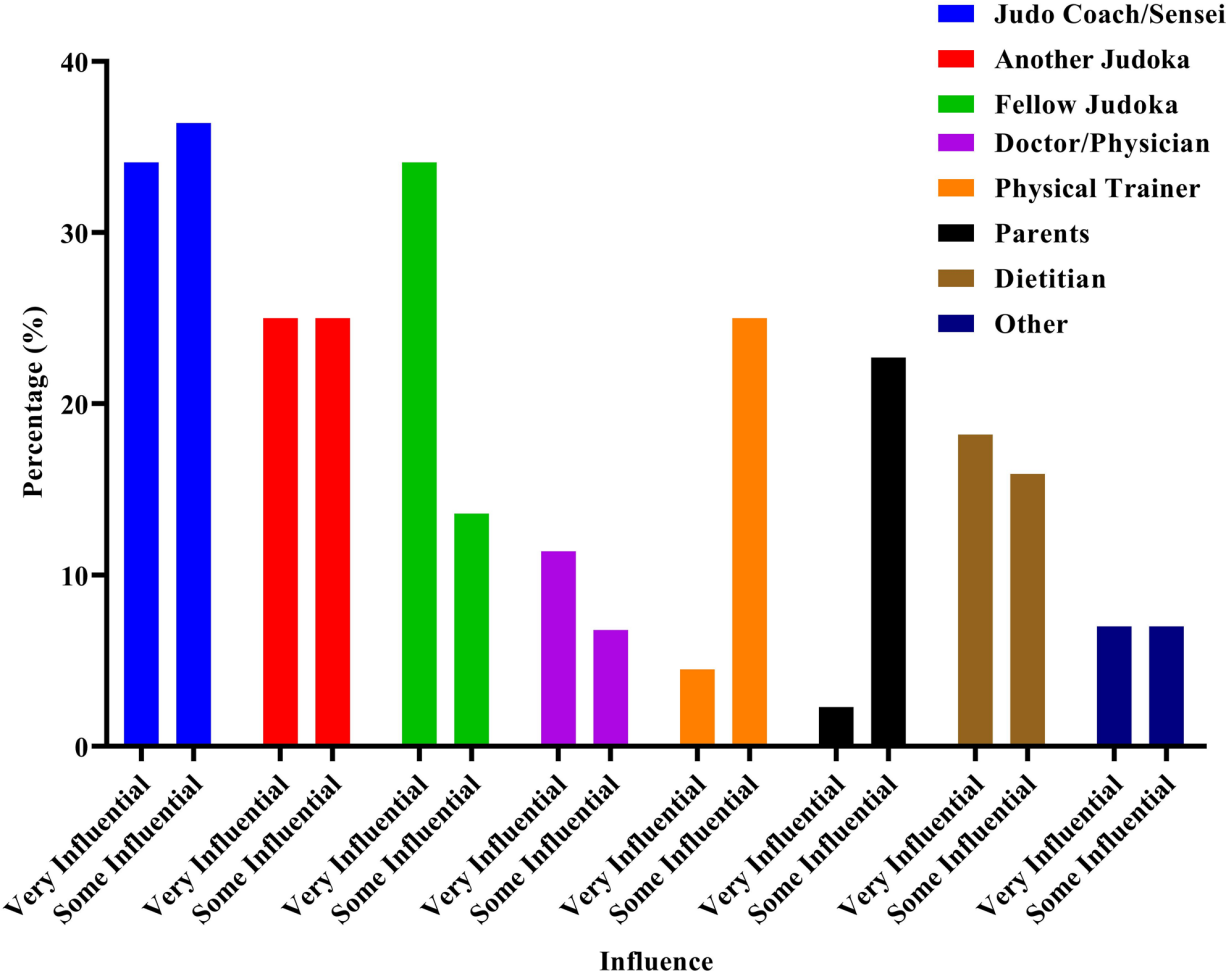
Frequency distribution for the source of influence

Participants reported being very influenced or somewhat influenced by their judo coach/sensei (70.5%), another judoka (50%), and peers in judo (47.7%). Most athletes were not influenced by doctors/physicians (56.8%), physical trainers (56.8%), parents (56.8%), or dietitians (45.5%) (See Fig. 2 and Tables in the supplementary file).

Spearman Rank-Order Correlation Analysis results are shown in Table 3. Age at which participants started practicing judo was negatively correlated with usual weight loss percentage (ρ=−0.337, *p* < 0.05, [weak]), and weight regain after 1 week of competition (ρ=−0.454, *p* < 0.01, [moderate]). Highest weight loss value was positively correlated with highest weight loss percentage (ρ = 0.916, *p* < 0.01, [strong]), usual weight loss value (ρ = 0.330, *p* < 0.05, [weak]), and weight regain after 1 week of competition (ρ = 0.406, *p* < 0.01, [moderate]). Highest weight loss percentage was positively correlated with usual weight loss percentage (ρ = 0.446, *p* < 0.01, [moderate]), and weight regain after 1 week of competition (ρ = 0.394, *p* < 0.01, [weak]). Analysis results indicated that usual weight loss value was positively correlated with usual weight loss percentage (ρ = 0.938, *p* < 0.01, [strong]), weight regain after 1 week of competition (ρ = 0.693, *p* < 0.01, [moderate]), and WL total score (ρ = 0.523, *p* < 0.01, [moderate]). Usual weight loss percentage was positively correlated with weight regain after 1 week of competition (ρ = 0,675, *p* < 0.01, [strong]), WL total score (ρ = 0.515, *p* < 0.01, [moderate]). There was a positive relationship between weight regain after 1 week of competition and WL total score (ρ = 0.411, *p* < 0.01, [moderate]).

**Table 3:** Spearman Rank-Order correlation analysis results

Spearman’s Rho (ρ) Correlation Coefficient	Age began practicing judo (years)	Highest WL value (kg)	Highest WL percentage (%)	Usual WL value (kg)	Usual WL percentage (%)	Weight regain after 1 week of competition (kg)	WL Total Score
Age began practicing judo (years)	1.000						
Highest WL value (kg)	−0.131	1.000					
Highest WL percentage (%)	−0.244	0.916**	1.000				
Usual WL value (kg)	−0.257	0.330*	0.306	1.000			
Usual WL percentage (%)	−0.337*	0.353	0.446**	0.938**	1.000		
Weight regain after 1 week of competition (kg)	−0.454**	0.406**	0.394**	0.693**	0.675**	1.000	
WL Total Score	−0.153	0.112	0.094	0.523**	0.515**	0.411**	1.000

*p < 0.05, **p < 0.01

## Discussion

This study provides the first detailed analysis of WL practices among the world’s top 50 judokas during the Olympic qualification cycle, revealing widespread use of pathogenic methods, significant gender differences in the magnitude of weight fluctuation, and a coaching environment that is the primary influence on these behaviors. The main outcomes of this study were (i) male judokas exhibited a greater habitual weight loss percentage, maximum weight loss values throughout their careers (both percentage and in kilograms), and higher weight regain after one week of competition compared to female judokas; (ii) the most favored weight loss methods included gradual dieting, meal skipping, increased exercise, sauna use, training with rubber/plastic suits, training in deliberately heated environments, fluid restriction, and fasting; (iii) judokas were influenced by their judo coaches, fellow judokas, and peers within the sport related to WL; (iv) the age at which individuals started judo practice correlated with their weight loss and regain percentages, that is, athletes who started their career earlier resort to more aggressive weight loss behavior.

According to the study results, habitual weight loss percentages, maximum career weight loss (both in percentage and kilos), and weight regain one-week post-competition values of male judokas were higher than female judokas. These results aligned with prior research indicating that female sambo athletes engage in more cautious weight loss practices compared to males [25]. Also, the research conducted by Zhong, Lakicevic [26] showed that the percentage of weight loss of male boxers were higher than females. Although significant differences were observed in some variables regarding the history of WL among female and male athletes, it was determined that the WL scores were similar. In the studies conducted by Ranisavljev, Kuzmanovic [27] and Artioli, Gualano [3] similar results were found in terms of WL scores. Similar observations in the literature point the interaction of socio-cultural and physiological factors influencing weight management methods, suggesting that while the amount of weight reduction may differ between genders, the prevalence of WL practices remains broadly similar. Despite these similarities, the nuanced differences in weight management across genders point the necessity of tailored interventions and educational programs, particularly concerning the potential health ramifications of aggressive weight cutting.

Judokas preferred gradual dieting, skipping meals, increased exercise, saunas, training in intentionally heated environments, training with rubber/plastic suits, fluid restriction, and fasting as weight loss methods. Many of these methods, particularly those involving dehydration, are associated with significant negative health consequences, including electrolyte imbalances, increased oxidative stress, and excessive strain on the cardiovascular and renal systems [28]. The literature indicates that several research have yielded comparable results to those obtained. Ranisavljev, Kuzmanovic [27] identified gradual dieting, increased exercise, and sauna usage as the key WL strategies employed in their study. In the research by Artioli, Scagliusi [9], athletes indicated that employing a mixture of hypohydration-inducing techniques (e.g., limited fluid consumption, training in plastic or rubberized suits under the judo outfit) increased exercise and reduced food intake. In the study conducted by Drid, Figlioli [25], the most effective weight loss strategies were gradual dieting, sauna use, and meal skipping. Recent research on Chinese amateur boxers [26] indicated that the most often employed tactics by the athletes were increased exercise, training in plastic suits, progressive dieting, training in heated environments, meal skipping, fluid restriction, and sauna use. A study on kickboxers [29] revealed that gradual dieting and increased exercises were the main strategies used by the athletes. A systematic review study conducted on combat sport athletes indicated that main weight loss methods employed by these athletes were increased exercise, gradual dieting [30]. These trends indicate a widespread dependence on traditional, but sometimes challenging approaches to attain competitive weight in combat sports. This method application uniformity highlights a shared understanding of weight-cutting practices across disciplines and different areas of the world, often driven by immediate performance goals rather than long-term health considerations. A deeper physiological inquiry is needed to elucidate how these conventional weight-loss strategies collectively influence athletic capability, holistic health, and the propensity for chronic detrimental effects. Furthermore, although these strategies are extensively used, their varying effects on male and female athletes, given fundamental physiological differences, necessitate further scrutiny.

A hierarchy of influence about the weight management guidance was observed, with coaches and peers in the judo community being the main sources of weight management guidance, as opposed to external health experts. In a study conducted on Chinese amateur boxers it was also found that the coaches and fellow athletes were the most significant source of influence [26], while health experts and scientific resources had very little impact. This critical finding underscores the urgent necessity of developing sport-specific internal networks that could offer safer and scientifically grounded medical and nutritional advice, thereby mitigating the well-documented health risks associated with rapid weight loss [28]. It can be inferred that coaches play a critical role in distributing essential information related to WL. Consequently, educational interventions targeting coaches are important for supporting healthier weight management behaviors among judokas. Furthermore, educating athletes about the physiological consequences of aggressive weight loss practices is vital for empowering evidence-based decisions.

Various WL parameters and age at judo practice onset were significantly correlated. Also, similar correlations were observed between weight loss and regain parameters. Especially, judo starting age and both usual weight loss percentage and weight regain one-week post competition values were inversely correlated. This may imply that athletes who begin judo training at a younger age develop more entrenched physiological or psychological coping mechanisms for managing body weight, potentially influencing their susceptibility to WL and subsequent regain. The fact that athletes start losing weight at a very early age may have normalized using dangerous weight management practices and led to more entrenched and severe behaviors in adulthood where they participate in numerous competitions throughout the year to qualify for the Olympics Games. Highest weight loss values and percentages were positively correlated with usual weight loss and regain following a competition. This situation indicates a systemic weight fluctuation pattern that strengthens with early weight loss attempts. When these findings evaluated as a whole chronic nature of weight cycling in judo and its potential physiological effects over time are highlighted which necessitates further examination into adaptive processes to weight fluctuations of the athletes. In order to compete in specific weight categories a considerable number of judokas use WL methods [31] which cause the athletes to experience suboptimal hydration during competition and training sessions [32]. The observed statistical correlations in this research point the widespread usage of WL methods within judo. Further research is needed to mitigate its potential negative effects on judo athletes’ overall health and performance.

## Conclusions

The study results provide significant insights into prevalent weight management practices, influencing factors, and gender-specific disparities among elite judokas, emphasizing areas where focused educational and intervention initiatives could significantly enhance athlete well-being and performance. The widespread reliance on pathogenic weight-loss methods, which can induce detrimental physiological states such as electrolyte imbalance, oxidative stress, and cardiovascular strain [28], coupled with the finding that coaches and peers are the primary sources of guidance, leads to a central and critical recommendation. The educational interventions could involve integrating sport dietitians into national teams to monitor athletes’ weight loss process closely, creating mandatory workshops for coaches related to physiology of weight management and developing educational resources for athletes about performance and health consequences of WL. Subsequent research ought to focus on longitudinal studies to assess the long-term health effects of repeated WL cycles, alongside the efficacy of multidisciplinary interventions involving sports nutritionists and exercise physiologists in promoting sustainable weight management practices. Also, including a wider range of judokas, not just the elite top 50, could provide greater understanding of how weight loss behaviors change at different levels of competition and stages of development.

### Study limitations

Although this study provides valuable insights, it has its limitations. First, the cross-sectional design offers a snapshot of WL practices at a single point in time and cannot establish causality or track the evolution of these behaviors throughout an athlete’s career or the Olympic qualification cycle. Therefore, longitudinal studies are suggested with the official support of umbrella organizations. Second, data were collected using a self-report questionnaire, which is susceptible to recall bias and social desirability bias. Athletes may not have fully reported the utilization of rigorous weight control methods. Therefore, objective measurements such as weighing athletes at the official weigh-in and next day can be suggested. Third, the sample size (*N* = 44), while consisting of a highly specialized and hard-to-reach group of world-class athletes, may restrict the statistical power and generalizability of the results. The sample was predominantly male (72.7%), perhaps influencing the validity of the gender-based comparisons. Fourth, the study took place at just one major event, the 2024 Antalya Grand Slam. Athletes’ WL behaviors may fluctuate based on the significance of the event, their standing in the qualifying cycle, or the timing within the competitive season, aspects that this study did not address. Finally, the questionnaire evaluated the techniques and extent of weight loss; however, it lacked objective physiological indicators (e.g., hydration status, body composition, health biomarkers) to validate the self-reported information or to quantify the specific physiological effects of the reported practices.

Future research would benefit from a longitudinal, multi-competition design that integrates objective physiological measurements to corroborate self-reported data and further the understanding of the health and performance ramifications of WL in this elite population.

## Supplementary Information

Supplementary Material 1

Supplementary Material 2

## Data Availability

The datasets used in the current study are available from the corresponding author on reasonable request.
