# Supplementary material for: Weight loss magnitude, prevalence and methods among male and female Olympic-level judo athletes
Source: BMC Sports Sci Med Rehabil. 2025 Dec 16;17:385. doi: 10.1186/s13102-025-01478-8 (PMC12750899; doi:10.1186/s13102-025-01478-8)
Supplement: Supplementary file 1 — Supplementary Material 1 [file 13102_2025_1478_MOESM1_ESM.docx]

*Frequency Distribution (%) of the Weight Loss Methods Preferred by Judokas*

| **Methods** | **Always** | **Sometimes** | **Rarely** | **Never Used** | **I Don’t Use Anymore** |
| --- | --- | --- | --- | --- | --- |
| **Gradual Dieting (%)** | **56.8** | **31.8** | **0.0** | **11.4** | **0.0** |
| Male | 40.9 | 27.3 | 0.0 | 4.5 | 0.0 |
| Female | 15.9 | 4.5 | 0.0 | 6.8 | 0.0 |
| **Skipping 1 or 2 Meals (%)** | **18.2** | **59.1** | **18.2** | **0.0** | **4.5** |
| Male | 15.9 | 38.6 | 15.9 | 0.0 | 2.3 |
| Female | 2.3 | 20.5 | 2.3 | 0.0 | 2.3 |
| **Fasting (%)** | **15.9** | **36.4** | **25.0** | **18.2** | **4.5** |
| Male | 11.4 | 31.8 | 11.4 | 15.9 | 2.3 |
| Female | 4.5 | 4.5 | 13.6 | 2.3 | 2.3 |
| **Restricting Fluid Ingestion (%)** | **31.8** | **25.0** | **20.5** | **13.6** | **9.1** |
| Male | 27.3 | 15.9 | 20.5 | 4.5 | 4.5 |
| Female | 4.6 | 9.1 | 0.0 | 9.1 | 4.5 |
| **Increased Exercises (%)** | **43.2** | **29.5** | **18.2** | **9.1** | **0.0** |
| Male | 29.5 | 20.5 | 13.6 | 0.0 | 0.0 |
| Female | 13.6 | 9.1 | 4.5 | 9.1 | 0.0 |
| **Training Intentionally in Heated Rooms (%)** | **34.1** | **25.0** | **18.2** | **20.5** | **2.3** |
| Male | 29.5 | 15.9 | 11.4 | 13.6 | 2.3 |
| Female | 4.5 | 9.1 | 6.8 | 6.8 | 0.0 |
| **Saunas** | **27.3** | **43.2** | **22.7** | **2.3** | **4.5** |
| Male | 22.7 | 29.5 | 15.9 | 2.3 | 2.3 |
| Female | 4.5 | 13.6 | 6.8 | 0.0 | 2.3 |
| **Training With Rubber/Plastic Suits (%)** | **45.5** | **22.7** | **18.2** | **6.8** | **6.8** |
| Male | 43.2 | 13.6 | 4.5 | 6.8 | 4.5 |
| Female | 2.3 | 9.1 | 13.6 | 0.0 | 2.3 |
| **Using Winter or Plastic Suits Whole day (%)** | **27.3** | **2.3** | **29.5** | **34.1** | **6.8** |
| Male | 22.7 | 2.3 | 13.6 | 29.5 | 4.5 |
| Female | 4.5 | 0.0 | 15.9 | 4.5 | 2.3 |
| **Spitting (%)** | **0.0** | **13.6** | **15.9** | **59.1** | **11.4** |
| Male | 0.0 | 9.1 | 13.6 | 15.9 | 6.8 |
| Female | 0.0 | 4.5 | 2.3 | 43.2 | 4.5 |
| **Laxatives (%)** | **4.5** | **29.5** | **13.6** | **52.3** | **0.0** |
| Male | 4.5 | 13.6 | 9.1 | 45.5 | 0.0 |
| Female | 0.0 | 15.9 | 4.5 | 6.8 | 0.0 |
| **Diuretics (%)** | **2.3** | **18.2** | **20.5** | **59.1** | **0.0** |
| Male | 2.3 | 13.6 | 13.6 | 43.2 | 0.0 |
| Female | 0.0 | 4.5 | 6.8 | 15.9 | 0.0 |
| **Diet Pills (%)** | **2.3** | **13.6** | **29.5** | **54.5** | **0.0** |
| Male | 2.3 | 11.4 | 13.6 | 45.5 | 0.0 |
| Female | 0.0 | 2.3 | 15.9 | 9.1 | 0.0 |
| **Vomiting (%)** | **2.3** | **18.2** | **25.0** | **54.5** | **0.0** |
| Male | 2.3 | 15.9 | 11.4 | 43.2 | 0.0 |
| Female | 0.0 | 2.3 | 13.6 | 11.4 | 0.0 |

*Frequency Distribution for the Source of Influence*

| **Source of Influence** | **Very Influential** | **Some Influential** | **Unsure** | **Little Influential** | **Not Influential** |
| --- | --- | --- | --- | --- | --- |
| **Another Judoka (%)** | **25.0** | **25.0** | **4.5** | **18.2** | **27.3** |
| Male | 22.7 | 15.9 | 4.5 | 11.4 | 18.2 |
| Female | 2.3 | 9.1 | 0.0 | 6.8 | 9.1 |
| **Fellow Judoka (%)** | **34.1** | **13.6** | **9.1** | **13.6** | **29.5** |
| Male | 25.0 | 9.1 | 9.1 | 9.1 | 20.5 |
| Female | 9.1 | 4.5 | 0.0 | 4.5 | 9.1 |
| **Doctor/Physician (%)** | **11.4** | **6.8** | **9.1** | **15.9** | **56.8** |
| Male | 9.1 | 4.5 | 4.5 | 15.9 | 38.6 |
| Female | 2.3 | 2.3 | 4.5 | 0.0 | 18.2 |
| **Physical Trainer (%)** | **4.5** | **25.0** | **2.3** | **11.4** | **56.8** |
| Male | 2.3 | 18.2 | 2.3 | 9.1 | 40.9 |
| Female | 2.3 | 6.8 | 0.0 | 2.3 | 15.9 |
| **Judo Coach/Sensei** | **34.1** | **36.4** | **4.5** | **15.9** | **9.1** |
| Male | 18.2 | 27.3 | 4.5 | 13.6 | 9.1 |
| Female | 15.9 | 9.1 | 0.0 | 2.3 | 0.0 |
| **Parents (%)** | **2.3** | **22.7** | **4.5** | **13.6** | **56.8** |
| Male | 2.3 | 6.8 | 2.3 | 11.4 | 50.0 |
| Female | 0.0 | 15.9 | 2.3 | 2.3 | 6.8 |
| **Dietitian (%)** | **18.2** | **15.9** | **6.8** | **13.6** | **45.5** |
| Male | 13.6 | 11.4 | 4.5 | 13.6 | 29.5 |
| Female | 4.5 | 4.5 | 2.3 | 0.0 | 15.9 |
| **Other (%)** | **7.0** | **7.0** | **4.7** | **2.3** | **79.1** |
| Male | 4.7 | 4.7 | 2.3 | 0.0 | 60.5 |
| Female | 2.3 | 2.3 | 2.3 | 2.3 | 18.6 |
