# Supplementary material for: Weight loss magnitude, prevalence and methods among male and female Olympic-level judo athletes
Source: BMC Sports Sci Med Rehabil. 2025 Dec 16;17:385. doi: 10.1186/s13102-025-01478-8 (PMC12750899; doi:10.1186/s13102-025-01478-8)
Supplement: Supplementary file 2 — Supplementary Material 2 [file 13102_2025_1478_MOESM2_ESM.pdf]

Appendix 2

Questionnaire about pre-competition rapid weight loss
Answer the questions with as much **attention** and **seriousness** as possible.
THE QUESTIONS REFERS TO RAPID WEIGHT LOSS IN ORDER TO COMPETE IN A GIVEN WEIGHT CLASS
The University of Sao Paulo thanks for your participation!

GENERAL INFORMATION. **Today's Date: _____/_____/_______.**

1. Age: ________ years.

2. Gender: ( ) male ( ) female

3. At what age did you begin to **practice** judo? ___________ years.

4. At what age did you begin to **compete** judo? ___________ years.

5. How much do you weight? ________ kg.

6. How tall are you? ________ m.

7. Please describe your achievements and participation in Judo competitions to date:

Regional or city level competition (*give some examples of regional or city level competitions*)
( ) participated without winning medal ( ) won a medal ( ) never participated

State level (*give some examples of state level competitions*)
( ) participated without winning medal ( ) won a medal ( ) never participated

National level (*give some examples of national level competitions*)
( ) participated without winning medal ( ) won a medal ( ) never participated

International level (*give some examples of international level competitions*)
( ) participated without winning medal ( ) won a medal ( ) never participated

8. How many times did you compete in the last year (including non-official competitions)?___________________.

9. In how many competitions did you win medal in the last year (including non-official competitions)?___________.

WEIGHT HISTORY AND DIET PATTERNS.

10. In which weight class do you compete? under ___________kg.

11. Did you change your weight class in the last two years?
( ) yes. In which weight classes did you compete?____________.
( ) no, I competed in the same weight class in the last two years

12. How much did you weigh in the last judo off-season *(especify the year)* ? _________ kg.

13. Have you ever lost weight in order to compete?
( ) Yes. (please continue aswering the rest of the questionnaire)
( ) No, I have never cut weight to compete (thank you for your help - do not answer the following questions).

14. What is the MOST WEIGHT that you have cut to compete in your career? _____________ kg.

15. How many times did you cut weight to compete last season *(especify the year)*?_____________ times.

16. How much weight do you **usually** cut before competitions? ______________ kg.

17. In how many days do you **usually** cut weight before competitions? ________________ days.

18. At what age did you begin to cut weight for competitions? _____________ years old.

19. How much weight do you usually regain in the week following a competition?___________ kg/week.

please continue on the next page

16000838, 2010, 1, Downloaded from https://onlinelibrary.wiley.com/doi/10.1111/j.1600-0838.2009.00940.x by Kastamonu University, Wiley Online Library on [30/11/2025]. See the Terms and Conditions (https://onlinelibrary.wiley.com/terms-and-conditions) on Wiley Online Library for rules of use; OA articles are governed by the applicable Creative Commons License

20. Using the scale below, please rate the amount of influence that each individual listed below has had on your weight loss practices. (i.e.: who encouraged and taugth you to lose weight) (check all items)

1	2	3	4	5
not influential	little influential	unsure	some influential	very influential

( ) another judoka/training colleague
( ) fellow judoka;
( ) physician/doctor;
( ) physical trainer;
( ) judo coach/ sensei;
( ) parents;
( ) dietitian;
( ) other. Explain ____________

21. The table below presents several methods to lose weight rapidly. Using the table below, HOW OFTEN did you use each one of the following methods to lose weight **before competitions**? (Check all items).

**Gradual dieting (lose weight in 2 weeks or more)**	always ( )	sometimes ( )	almost never ( )	never used ( )	I don't use anymore ( )
**Skipping 1 or 2 meals**	always ( )	sometimes ( )	almost never ( )	never used ( )	I don't use anymore ( )
**Fasting (not eating all day)**	always ( )	sometimes ( )	almost never ( )	never used ( )	I don't use anymore ( )
**Restricting fluids ingestion**	always ( )	sometimes ( )	almost never ( )	never used ( )	I don't use anymore ( )
**Increased exercises (more than usual)**	always ( )	sometimes ( )	almost never ( )	never used ( )	I don't use anymore ( )
**Training intentionally in heated training rooms**	always ( )	sometimes ( )	almost never ( )	never used ( )	I don't use anymore ( )
**Saunas**	always ( )	sometimes ( )	almost never ( )	never used ( )	I don't use anymore ( )
**Training with rubber/plastic suits**	always ( )	sometimes ( )	almost never ( )	never used ( )	I don't use anymore ( )
**Use winter or plastic suits during the whole day and/or night (without exercising)**	always ( )	sometimes ( )	almost never ( )	never used ( )	I don't use anymore ( )
**Spitting**	always ( )	sometimes ( )	almost never ( )	never used ( )	I don't use anymore ( )
**Laxatives**	always ( )	sometimes ( )	almost never ( )	never used ( )	I don't use anymore ( )
**Diuretics**	always ( )	sometimes ( )	almost never ( )	never used ( )	I don't use anymore ( )
**Diet pills**	always ( )	sometimes ( )	almost never ( )	never used ( )	I don't use anymore ( )
**Vomiting**	always ( )	sometimes ( )	almost never ( )	never used ( )	I don't use anymore ( )

16000838, 2010, 1, Downloaded from https://onlinelibrary.wiley.com/doi/10.1111/j.1600-0838.2009.00940.x by Kastamonu University, Wiley Online Library on [30/11/2025]. See the Terms and Conditions (https://onlinelibrary.wiley.com/terms-and-conditions) on Wiley Online Library for rules of use; OA articles are governed by the applicable Creative Commons License

Appendix 3

Scores:

Question 13: yes = 3 points; no = 0 points
Question 14 = 0.5 points per kg
Question 15 = 1 point per time
Question 16 = 1 point per kg
Question 17:
1–3 days = 5 points
4–5 days = 4 points
6–7 days = 3 points
8–10 days = 2 points
11–14 days = 1 point
15 days or more = 0 points
Question 18:
14 years or less = 5 points
15 years = 4 points
16 years = 3 points
17 years = 2 points
18 years = 1 point
19 years or more = 0 points
Question 19 = 1 point per kg
Question 21:
always = 3 points per method
sometimes = 2 points per method
almost never = 1 point per method
never used = 0 points
I don't use anymore = 0.5 point per method
"gradual dieting" and "increased exercising" = 0 points
"laxatives", "diuretics", "diet pills" and "vomiting" = frequency score × 2

16000838, 2010, 1, Downloaded from https://onlinelibrary.wiley.com/doi/10.1111/j.1600-0838.2009.00940.x by Kastamonu University, Wiley Online Library on [30/11/2025]. See the Terms and Conditions (https://onlinelibrary.wiley.com/terms-and-conditions) on Wiley Online Library for rules of use; OA articles are governed by the applicable Creative Commons License
